# Nudging societally relevant behavior by promoting cognitive inferences

**DOI:** 10.1038/s41598-022-12964-1

**Published:** 2022-06-02

**Authors:** Pieter Van Dessel, Yannick Boddez, Sean Hughes

**Affiliations:** Department of Experimental-Clinical and Health Psychology, Ghent University, 9000 Ghent, Belgium

**Keywords:** Psychology, Human behaviour, Psychology and behaviour

## Abstract

Effective behavioral interventions are essential to address urgent societal challenges. Over the past decade, nudging interventions (i.e., arranging the environment to promote adaptive behavioral choices) have surged in popularity. Importantly, effective application of the nudging approach requires clear guiding principles with a firm basis in behavioral science. We present a framework for nudging interventions that builds on evidence about the goal-directed inferential processes underlying behavior (i.e., processes that involve context-dependent inferences about goals and the actions available to achieve these goals). We used this framework to develop nudging interventions that target context-relevant cognitive inferences. We examined the effectiveness of these inference nudging interventions for promoting two important types of societal behavior: pro-environmental actions and adherence to COVID-19 guidelines. As predicted, two online studies revealed that inference nudging interventions successfully increased energy conservation (Study 1) as well as social distancing during the COVID-19 crisis (Study 2). A field experiment found that inference nudging interventions increased hand disinfection in a real-life store during the COVID-19 crisis (Study 3). Our findings highlight the importance of applying state-of-the-art insights about the (inferential) determinants of behavior in behavior change interventions.

## Introduction

Adaptive human behavior is instrumental if we are to reach key objectives both at the individual and societal level. Achieving these objectives requires interventions that effectively shape behavior when it is suboptimal, whether that involves improving people’s mental^1^ or physical health^2^, combatting societal threats such as pandemics^3–5^ or larger scale issues such as global warming^6–8^. The importance of effective interventions to promote adaptive individual and societally relevant behavior cannot be overstated^9^.

In contrast to more traditional (legislation or financial incentive) interventions to promote adaptive behavior, ‘nudging’ interventions have been introduced to promote adaptive behavioral choices (i.e., choices to engage in behavior that has proven benefits for the individual or their environment, such as buying healthy foods) without restricting freedom of choice or providing economic incentives (e.g., by placing healthy food within arm’s reach)^10,11^. These interventions typically involve small changes to natural choice environments and have often been found to be effective and beneficial for society and individuals when compared to traditional policy tools that are often more costly and difficult to implement^12–15^. As a direct result, the popularity of nudging interventions has exploded and they are widely applied in the lab by researchers and in the wider world by policy-makers^9,11^.

While there is little doubt about the value of the nudging approach in general, it has also become clear that the nudging approach is not a silver bullet solution. Most importantly, effect sizes of nudging interventions vary considerably^15^ and several nudging interventions have proven to be ineffective^16,17^ or to backfire (approximately 15% of interventions^15^). Effects are also typically tied to the context in which the nudge is provided with little generalization across time or context^18^. These limitations at the empirical level are not surprising. Nudging has been defined as “any aspect of the choice architecture that alters people’s behavior *in a predictable way* without forbidding any options or significantly changing their economic incentive”^11^. However, predicting (changes in) behavior is not an easy task and (intuitive) ideas about the effect of environmental changes on behavior can be inaccurate. For instance, a recent large-scale study tested the effectiveness of 22 nudges to encourage vaccination and found that neither intervention scientists nor laypeople correctly predicted the top-performing intervention^19^.

To foster more successful application of the nudging approach, clear guiding principles for nudging interventions based on scientific evidence are required^20^. Notably, predicting (and influencing) behavior is the primary aim of behavioral science. Yet, while early work on nudging initially set out to apply behavioral science^11^, interventions often lack the integration of scientific insights about the determinants of behavior^18,21^. To re-align nudging interventions with this aim, we present a novel framework for nudging interventions that builds on evidence about the mental processes that underlie behavior.

**Figure 1:**
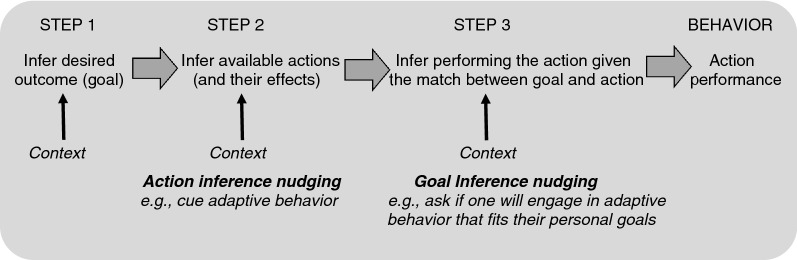
Illustration of the inferential process underlying behavior. Internal or external contextual influences first promote inference of a wanted outcome or goal. For instance, a person who sees other people in a store may infer that they would like to keep themselves and others safe from COVID-19. Next, an inference is made about the availability of certain actions and its expected effects. For instance, when seeing an alcohol dispenser with a sign to disinfect hands, a person may infer that they can disinfect their hands to adhere with COVID-19 guidelines. Step 3 involves the inference of performing a certain action given the match between expected action effects and current goals. For instance, a person may infer that they will disinfect their hands given the match with their goal to keep themselves and others safe from COVID-19. The latter inference may (automatically) translate into action performance (e.g., hand disinfection). Contextual influences moderate each of the inference steps. Action inference nudging interventions specifically target Step 2 whereas goal inference nudging interventions target Step 3.

Over the past several decades, two mental processes have emerged as powerful determinants of behavior: goals^22,23^ and belief-based inferences^24^. Indeed, there is a growing consensus in cognitive (neuro)science that all behavior is based on context-dependent inferences about desired outcomes (i.e., goals) and the actions available to achieve these goals^25,26^. In an inferential framework, the idea is that contextual influences may promote inferences of a desired outcome or goal (Step 1), of available actions and its effects (Step 2), and of performing an action given the match between expected action effects and current goals (Step 3), which determines action performance (Fig. 1). This framework can be used to optimize nudging interventions so that goals and inferential processes are the direct target of the intervention’s design.

To provide an initial test of the value of this inferential nudging approach, we carried out three pre-registered studies to investigate the effectiveness of interventions that target these inferential processes to promote two types of behavior of societal importance: pro-environmental behavior and adherence to COVID-19 guidelines. In each study, we used the inferential framework in two ways to develop inference nudging interventions adapted to the choice context. First, we developed an ‘action inference nudging’ intervention that targets inferences about the availability of adaptive behavior (and its effects) (Step 2). Second, we developed a ‘goal inference nudging’ intervention that targets inferences that one will perform adaptive behavior because it accords with personal goals (Step 3). Our main hypothesis was that all our nudging interventions would promote adaptive behavior. A secondary hypothesis was that the goal inference nudging interventions would be able to produce effects that generalize across contexts and over time because they promote self-agency inferences about the goal-directed behavior that one is likely to engage in. Enacting such inferences in one context can promote their application across time and in different contexts^27–29^.

## Results

### Study 1: Nudging pro-environmental behavior

Our sample consisted of 301 participants (UK residents, 171 women, *M*_*age*_ = 35.8, *SD* = 12.3). Participants performed a figure search task three times: before (baseline), just after/ during (immediate impact), and 24 h (continued impact) after a nudging intervention. In this task, they could move a slider to either increase or decrease the figure brightness level, and as a result, either increase or decrease their electrical expenditure. To create a goal conflict (and test if our nudging interventions can be effective in this situation), we told participants we would pay them more money when completing the task faster. Saving electricity (by reducing figure brightness) increased task difficulty and thus reduced their access to monetary rewards whereas wasting electricity had the opposite effect. Before and after the study, participants also reported whether they had engaged in real-life energy saving behavior in the previous 24 h and how often they had done so.

Participants were randomly assigned to one of three conditions: action inference nudging, goal inference nudging, or control. Before the second figure search task, the goal inference nudging group were asked to think about positive (and negative) consequences of choosing (not) to perform energy saving behavior and indicated whether, given these consequences, they would be more likely to perform energy saving or wasteful behavior. This intervention targets the Step 3 inference that one may engage in energy saving behavior because it helps them achieve desired outcomes. The action inference nudging and control groups received similar prompts about an unrelated topic (drug use). When completing the second figure search task, the action inference nudging group received a set of nudges to highlight energy saving behavior, such as a prompt to move the slider to be more environmentally friendly. This interventions targets the Step 2 inference that one can easily engage in energy saving behavior (which has environmentally friendly effects).

**Figure 2:**
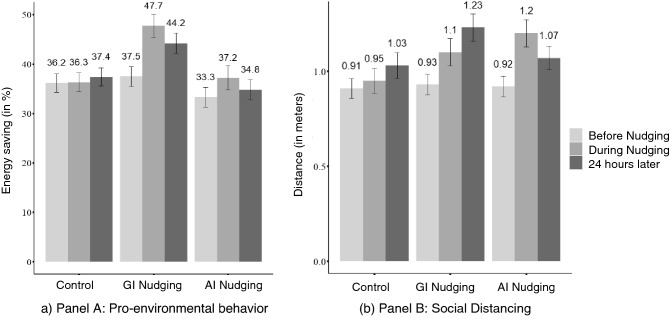
Indices of pro-environmental behavior (Study 1) and social distancing (Study 2) by Nudging Condition and Time. This figure displays the indices of pro-environmental behavior (reversed slider value −% energy saving in the figure search task) in Study 1 and of social distancing (distance from predecessor in the virtual shopping task) in Study 2. Indices are reported before nudging, during nudging, and 24 h later, in the control, goal inference (GI) nudging, and action inference (AI) nudging conditions. Error bars represent ± 1 standard error of the mean.

Consistent with our predictions, pro-environmental behavior (i.e., electricity saving) increased from baseline to immediate post-intervention in both nudging groups, *t*s > 1.81, *p*s < 0.037, *d*s > 0.18. This increase in pro-environmental behavior was still evident 24 h later in the goal inference nudging, *t*(93) = −3.68, *p* < 0.001, *d* = 0.38, but not the action inference nudging group. Compared to the control group, only the goal inference nudging group exhibited a significantly stronger increase in pro-environmental behavior from baseline, both when assessed immediately and a day later, *t*s > 2.03, *p*s < 0.022, *d*s > 0.29 (Fig. 2). Although there were no significant differences between groups in the self-reported amount of energy saving behavior in real life, the increase in the proportion of participants who reported having engaged in real-life energy saving behavior after the study was higher for the goal inference nudging compared to other groups, *t*s > 2.82*, p*s < 0.003*, d*s > 0.39. However, this result should be interpreted with caution because the proportion of participants who reported having engaged in energy saving behavior at baseline was higher for the goal inference nudging than for the control group, *χ *^*2*^(1) = 4.68, *p* = 0.030.

### Study 2: Nudging social distancing during COVID-19 lockdown

During the first COVID-19 lockdown in the UK (May 2020), we recruited 222 participants (UK residents, 125 women, *M*_*age*_ = 24.8, *SD* = 3.5) who had difficulty adhering to COVID-19 guidelines, and who had violated social distancing regulations during their last shopping experience. They completed a shopping task in a virtual store three times: before, during, and 24 h after a nudging intervention.

Each trial required them to first select a specific item in the store, queue in-line at the check-out, and to select the distance they wanted to keep from the next person in the queue. Participants were told they should treat this store as if it were a real-life store during COVID-19 lockdown. In-store prompts indicated that customers should maintain at least 2 m distance from other customers due to the COVID-19 crisis. We created a goal conflict by telling participants we would pay them more money for completing their virtual shopping faster. During practice trials, participants subsequently learned that maintaining larger distances led to other customers cutting in line before them, leading to longer trial durations, and less money. Before and after the study, participants also reported distance violations they had made when shopping in real-life in the previous 24 h.

During the second shopping task, the goal inference nudging group encountered a poster in the virtual store depicting an old couple and the text: “One person not keeping their distance can lead to the death of hundreds of grandparents, like your own. Make your choice. What do you choose to do, keep your distance or not?”. This intervention targets the Step 3 inference that one may maintain distance because this matches one’s goal of have healthy grandparents (which was established as an important goal during pre-screening). The action inference nudging group encountered a virtual store with crosses and circles on the floor, highlighting where customers should stand in the queue to maintain 2 m distance. This intervention targets the Step 2 inference that one can select a distant spot (and adhere with COVID guidelines).

Consistent with our predictions, social distancing increased from baseline to immediate post-intervention in both nudging groups, *t*s > 3.34, *p*s < 0.001, *d*s > 0.40, and this increase was larger relative to the control group, *t*s > 2.16, *p*s < 0.016, *d*s > 0.36. Participants in all groups maintained more distance after 24 h (continued impact) than in the first shopping task (baseline). Yet, the goal inference nudging group exhibited a larger increase than the control and action inference nudging group, *t*(142) = 2.44, *p* = 0.008, *d* = 0.41 (Fig. 2). Self-reported real-life distance violations were also significantly lower when people had encountered goal inference nudging, compared to the other groups, *ts* > 1.72, *ps* < 0.044, *ds* > 0.28.

### Study 3: Nudging hand disinfection during COVID-19 lockdown

During the second COVID-19 lockdown in Belgium (February 2021), hand sanitizing behavior was observed of all customers of a Belgian grocery store for three weekdays (total N = 2198). We observed whether participants did or did not use hand disinfection at two locations in the store: at the entrance (where an alcohol dispenser and nudging signs were present) and the fresh foods area (where an alcohol dispenser was present but nudging signs were not; this allowed us to test for generalization).

Action inference nudging involved placing the alcohol dispenser very close to the entrance door along with a red sign next to the dispenser stating “please disinfect hands”. This intervention targets Step 2 inferences about the availability of this action and its effect of adhering with regulations. Goal inference nudging involved a similar situation with the sign changed to say ‘Disinfecting hands saves lives. Will you disinfect your hands?’ along with two posters of elderly and vulnerable people next to the dispenser repeating this message. This intervention targets the Step 3 inference that disinfecting one’s hands fits with a (personal) goal to protect the elderly and vulnerable. All nudges were absent for the control group.

**Figure 3:**
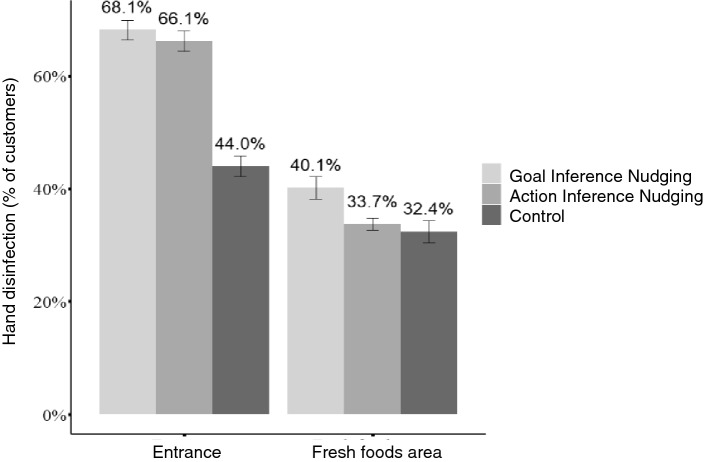
Hand disinfection in Study 3 at the store entrance (nudging site) and fresh foods area (generalization site) by condition. This figure displays the proportion of customers disinfecting their hands in each condition. Error bars represent ± 1 standard error of the proportion.

Consistent with our predictions, the proportion of participants using hand disinfection at the store entrance was higher for the goal (68.1%) and action inference nudging (66.1%) than the control group (44.0%), *p*s < 0.001, ORs > 1.50. These effects generalized to the fresh foods area, where sanitization was higher following goal (40.1%) than action inference nudging (33.7%) or controls (32.1%), *p*s < 0.013, ORs > 1.20 (Fig. 3). The average amount of used alcohol per customer entering the fresh foods area was higher in the goal inference nudging condition (0.48 g) compared to the other conditions (0.30–0.34 g), *p*s < 0.016, *d*s > 2.76.

## Discussion

Given the important role of behavior in key societal challenges, there is a strong need for effective, low-cost, and scalable behavioral interventions that make changes to natural choice environments (i.e., nudges). Understanding when and how nudging interventions should (and should not^30,31^) be used requires input from the behavioral sciences^18,32^, and in particular, a better understanding of the mental processes driving those effects. In light of recent calls to develop guiding principles for nudging interventions^20^, we present an inferential framework that can be used to build nudging interventions that target the goals^27,33^ and inferences^25,34^ people make in a choice environment. We used this framework to develop nudging interventions that target inferences about adaptive actions and its effects (Step 2 action inferences) and inferences that one will perform adaptive actions that fit important goals (Step 3 goal inferences). Overall inference nudging interventions increased adaptive behavior in the intervention context even in light of conflicting (e.g., monetary) goals. Interventions that targeted Step 3 inferences also showed generalization effects from the intervention context to other contexts.

Note that the general approach to nudge cognitive inferences may seem at odds with earlier approaches to nudging, and in particular, with those built on a dual-systems framework which distinguished between automatic and controlled mental processes^35,36^. The assumption behind those interventions was that (simple) nudges should target (simple) automatic processes (an approach that also led to some discussion about its ethicality^37^). These automatic processes were often thought not to be belief-based or inferential, but what exactly they were, and in what sense they were ‘automatic’, was not clearly defined^38,39^. Moreover, the strict separation between automatic and controlled processes and the mapping of inferential processes onto non-automaticity forwarded by the dual systems framework has long been criticized^39–41^.

The inferential approach to nudging sets this problematic distinction to the side and instead targets inferential processes irrespective of their automaticity features. This approach fits well with recent theorizing and empirical evidence on the mental processes underlying behavior^24–26^. It also builds on the idea that, although nudging interventions may be simple at the procedural level (i.e., they arrange the choice environment in subtle ways) and at the behavioral level (i.e., they produce relatively small changes in behavior), this does not mean that what is happening at the cognitive level is also necessarily simple. The impact of nudging interventions on behavior can be mediated by a variety of (complex) cognitive processes and if the aim of these interventions is to promote adaptive behavior (in a predictable way), then it seems beneficial to develop nudging interventions that recruit those processes necessary to produce optimal nudging effects (e.g., by directing the cognitive inferences that people make).

In line with this idea, we found that nudging interventions that explicitly target evidence-based (cognitive) determinants of behavior can lead to changes in societally relevant actions, from pro-environmental energy saving, to COVID-19 related distancing and sanitization. Note, however, that all the tested interventions were specifically designed to promote important types of behavior in specific samples (e.g., participants that have difficulty adhering to COVID-19 guidelines) and specific contexts (both online and in real-life). Our studies should not be taken to imply that observed effects of these interventions are widely generalizable. For instance, in Study 1, the energy-saving behavior was rather artificial and participants may not have considered this behavior to have a strong impact on the environment. Similarly, in Study 2, maintaining a safe distance had negative effects due to other customers cutting queue which might not occur in certain real world contexts. As a result, other inferences (such as inferences related to responding in a way desired by the experimenter) may contribute more or less strongly to energy-saving behavior or social distancing in other contexts or in other (e.g., less restrictive) samples. Crucially, however, the aim of our study was to provide an initial test of the value of our inferential framework for the development of nudging interventions. Our results support its value in experimental task contexts and in a real-life context which precludes a mere explanation in terms of desirability inferences (Study 3). Interventionists can use the inferential framework to consider what inferences to target within their specific context and target sample to develop a targeted nudging intervention (for which the effectiveness may then be tested within this context and sample).

Others have recently also sought to nudge in ways that target more elaborate (goal-directed) cognitive processes rather than behavior directly. For instance, nudge plus interventions^42^ combine typical nudges with an element of self-reflection that targets deliberative processes. Boosting interventions^27^ and autonomy-enhancing paternalism interventions^43^ target processes that promote the competence to engage in behavior that accords more with people’s goals. While nudging plus interventions only target controlled processes, boosting interventions also target (heuristic) inferences and are therefore more similar to (goal) inference nudging. Yet, boosting (and other) interventions are not grounded in a theoretical framework that explicates the different inferential steps driving behavior. Using this framework may aid the development of more successful interventions.

Similar to those recent approaches, inference nudging (and goal inference nudging in particular) also draws on personal goals and may therefore remove an oft raised ethical concern that nudging manipulates people into doing things that do not fit their goals^37^. Inference nudging therefore aligns well with early approaches to nudging that target behavior that accords with people’s own goals (pro-self nudges^10,44^). Notably, however, inference nudging may also present a new tactic to later nudging approaches that target pro-social behavior which does not always accord with people’s goals (pro-social nudges^44^). Specifically, (goal) inference nudging highlights a link between pro-social behavior and specific goals that a person may not have considered or be readily aware of and may therefore allow them to infer that this behavior does fit with some of their personal goals. This type of nudging bears similarity to contingency management interventions albeit without presentation of a reinforcer, and to ABC-training where goal-directed inferences are practiced extensively^30^. Of course, this tactic requires that inference nudging interventions draw on universal goals^45^ or are targeted to audiences with specific known goals (see Study 2).

In conclusion, we agree with recent trends in clinical science^29,46,47^ that an integrative effort is required for adequate behavior intervention, one that builds on state-of-the-art insights from different fields of behavioral science^48^. To live up to its full potential, future (inference-based) nudging interventions should take into account evidence about cognitive, but also social, environmental, and motivational determinants of behavior (e.g., about the goals people tend to pursue^45^). While more research on these determinants is (and will remain) crucial, our results highlight that evidence-based nudging interventions can already be an effective means to promote behavior of societal importance. Indeed, if the effect sizes observed in Study 3 are correct, then minor revisions to a shopping environment can, for instance, increase the number of people disinfecting their hands by 24 percentage points (55% relative increase) at the intervention site and 8 percentage points (25% relative increase) at other places. We hope that interventions informed by state-of-the-art behavioral insights can become a key tool in the arsenal of policy makers and organizations striving for societal benefit.

## Methods

### Ethics approval

This research complied with all relevant ethical regulations. All studies were conducted under approval of the Ethics Committee at Ghent University (reference number 2019/72, 2020/52, 2020/165). For the online studies (Study 1 and 2), informed consent was obtained from all participants as part of the enrollment process. All images in the online study and in the Supplementary Material figures were obtained from third party material for which we have permission for reuse.

### Study 1—Participants

We recruited 350 participants via Prolific Academic to have sufficient power to detect a between-subjects effect of *d* = 0.40 in a planned between-subjects *t*-test. We excluded the data from 49 participants who did not fully complete all questions and tasks or indicated issues when performing the study, leaving 301 participants (UK residents, 171 women, *M*_*age*_ = 35.8, *SD* = 12.3). For this and all other studies, prior to data-collection, target sample size was pre-registered together with the study design, study hypotheses, and data-analytic plans.

### Study 1—Design

In line with standard recommendations to prevent selective attrition^49^, participants were first (1) informed about the study duration and the requirement to complete different phases and (2) asked to do their best to complete all tasks in a thoughtful manner without taking a break to help facilitate scientific progress. Participants were also asked not to complete the study if they were color blind. Participants then provided informed consent and indicated their age, gender, and identification number for payment purposes. Next, participants were asked whether, in the previous 24 h, they ever considered that they should try to limit their energy consumption such as by conserving electricity (response options: yes/no) and on how many separate occasions they acted in accordance with this thought (e.g., thinking that closing the fridge or turning of the lights saves energy and actually doing so). They also indicated to what extent they found it personally important to (1) act environmentally friendly and (2) make as much money as possible in the studies they perform on Prolific Academic (see Table 1 for descriptives and randomization checks).

**Table 1:** Descriptives and randomization Check for Study 1. The table reports the average and standard deviations (in parentheses) of participant demographic variables and responses to questions about goals and prior energy saving behavior in each condition of Study 1, the p-value of the effect of condition in an ANOVA for each variable, and the number of participants in each condition. For the proportion variables (gender and proportion of participants reporting energy saving behavior), the p-value is for a Wald test. If a given p-value is greater than .05, it means the corresponding test does not allow us to reject the null hypothesis that all conditions have the same value for the corresponding variable.

	Goal inference nudging	Action inference nudging	Control	p-value
Age (years)	36.1 (12.1)	35.2 (10.2)	37.2 (13.0)	.51
Men	27.7%	32.6%	24.3%	.44
Goal to be pro-environmental	5.5 (1.4)	5.3 (1.5)	5.5 (1.4)	.53
Goal to make money	4.6 (1.8)	4.6 (1.7)	4.2 (1.8)	.65
Energy saving behavior figure search task 1	37.5 (19.7)	33.3 (19.3)	36.2 (19.6)	.31
Participants reporting energy saving behavior	60.6%	74.0%	75.7%	.043
Amount of energy saving behavior	2.7 (4.8)	2.4 (3.1)	2.8 (2.8)	.65
Number of participants	94	96	111	

Participants then received instructions about the figure search task. Participants were informed that they would need to find specific colored figure as quickly as possible and that they would receive more money than the default study payment if they would be faster than other participants in this task (for verbatim instructions see Supplementary Material and OSF link). Instructions then specified that the researchers are aware that presenting bright colors and large figures consumes a lot of electricity (a link was provided to a website supporting this claim) and that participants would therefore be allowed to decide for themselves how brightly they wanted to present the figures. To this end, they could move a slider to the left (and save more energy) or to the right (and save less energy). We explained that participants thus could serve two goals by use of the slider: to save more electricity but also make the task more difficult or to save less electricity but also make the task easier. After completing an instruction check, participants completed 8 practice trials in which they saw a display with 140 figures (circles and squares) in 4 different colors (blue, green, pink, brown) and they needed to click on one specific figure (e.g., a blue square) as quickly as possible. A slider was presented that participants could move left to display the figures less brightly and save more energy or to the right to display the figures more brightly (slider values 0–100%; slider start value = 50%). After clicking on the figure, participants were informed about their response time. After the practice phase, participants received the generic feedback that they were a little slower than the average participant who did this task before them. This was done to prevent potential confounding influences of differential motivation if participants would infer that they were likely faster (or slower) than others and would therefore receive the maximum (or minimum) payment. After receiving this feedback, participants completed 40 test trials.

After the first figure search task, participants in the goal inference nudging condition were informed that there are many harmful effects on the environment that result from wasting electricity. They were asked to list negative (positive) thoughts and consequences that would come to mind if they or others would choose energy wasteful (energy saving) behavior. Finally, participants selected what type of behavior they thought they would be more likely to choose to emit in the future given the consequences of energy saving and energy wasteful behavior they just stipulated themselves. This intervention builds on several well-supported intervention techniques established in (psychological) science^50–54^, to nudge the inference that they would engage in energy saving behavior because it helps them achieve desired outcomes. Participants in the control and action inference nudging condition performed the same task about an unrelated topic (drug use).

Participants then completed the same 40 test trials for the second figure search task with one exception. For participants in the action inference nudging condition, there were three changes. In accordance with other nudging interventions^55^: (1) a cue was presented: a large green arrow with the text: “Move slider to the left to be more environmentally friendly!”, (2) the slider value was presented in green/orange/red depending on the value, and (3) the default slider value was set lower (at 25% rather than 50% brightness) at the start of the task. Participants could then note any (visibility or personal) problems they had with the study and were reminded to come back for the second part of the study the following day.

The next day, participants first completed the same demographic questions and questions about real-life energy saving behavior as before and then completed the third figure search task (identical to the first figure search task). Next, participants were probed for demand compliance and reactance and were debriefed and thanked for their participation.

The main analyses constituted a 3 (Intervention Condition) × 3 (Time of Task Performance) mixed analysis of variance (ANOVA) on the mean figure search task slider values and real-life energy saving behavior and planned t-tests comparing differences in slider values and real-life energy saving behavior between conditions (Table 2).

**Table 2:** Means, SDs, and regression-estimated effects of condition for increase in pro-environmental behavior compared to baseline in Study 1. The table reports the average and standard deviations (in parentheses) of increase in pro-environmental behavior compared to baseline in Study 1, and the p-value of the effect of condition in an ANOVA for each variable.

	Goal inference nudging	Action inference nudging	Control	p-value
Energy saving behavior figure search task 2	10.2 (18.5)	3.9 (21.4)	0.1 (15.1)	< .001
Energy saving behavior figure search task 3	6.7 (17.6)	1.6 (17.1)	1.2 (17.7)	.050
Participants reporting energy saving behavior	10.6%	−9.4%	−9.0%	.014
Amount of energy saving behavior	−0.1 (5.2)	-0.6 (2.6)	−0.8 (2.7)	.35

### Study 2—Participants

In May 2020, during the first COVID-19 lockdown in the UK, we recruited 250 UK volunteers via Prolific Academic to allow sufficient power to find a between-subjects effect of *d* = 0.40. Invitation to the study was based on a pre-screening study in which the targeted participants had indicated that (1) they would go shopping later that day, (2) they would be available to complete the first study part before going shopping and the second part after going shopping, (3) the last time they went shopping was less than a week ago, (4) they had not kept their distance at least twice during the last time they went shopping, (5) they found it important that their grandparents would stay healthy, (6) they did not find it easy to adhere to COVID-19 guidelines, (7) they did not find it very important to follow COVID-19 guidelines, and (8) they found it important to make money on Prolific. We excluded the data of 28 participants who did not pass an attention check, leaving 222 participants (125 women, mean age = 25, *SD* = 4). Table 3 provides descriptives and randomization checks.

**Table 3:** Descriptives and randomization Check for Study 2*.* The table reports the average and standard deviations (in parentheses) of participant demographic variables and responses to questions about goals and social distancing behavior in each condition of Study 2, the p-value of the effect of condition in an ANOVA for each variable, and the number of participants in each condition. For gender, the p-value is for a Wald test. If a given p-value is greater than .05, it means the corresponding test does not allow us to reject the null hypothesis that all conditions have the same value for the corresponding variable.

	Goal inference nudging	Action inference nudging	Control	p-value
Age (years)	24.4 (3.8)	25.3 (3.5)	24.7 (3.4)	.25
Men	43.7%	50.0%	38.0%	.28
Goal to follow COVID-19 recommendations	5.3 (1.2)	5.3 (0.9)	5.2 (1.1)	.96
Goal to make money	4.8 (1.2)	4.7 (1.2)	5.0 (1.3)	.20
Goal to keep grandparents healthy	6.7 (0.6)	6.6 (0.6)	6.5 (0.6)	.31
Social distancing shopping task 1 (in meters)	0.9 (0.5)	0.9 (0.5)	0.9 (0.4)	.97
Social distancing thoughts real-life shopping	90.1%	84.9%	94.9%	.12
Social distancing real-life shopping	5.3 (4.5)	5.4 (3.6)	4.8 (3.7)	.68
Distance violations real-life shopping	5.2 (4.1)	4.8 (3.8)	4.6 (3.1)	.57
Number of participants	71	78	73	

### Study 2—Design

After informing participants about the duration of the study, they received instructions about the virtual shopping task. Participants were also asked to imagine that they themselves were shopping during the COVID-19 crisis and that they wanted to complete their shopping quickly. They were informed that performing this task faster than other participants would lead to a bigger monetary reward and that, each trial, they would first need to acquire a specific item in a virtual store by clicking on it and then queue at checkout to pay for the item. At checkout they would need to select where to stand in the queue for checkout by clicking on an open place but if they would choose to stand farther away from their predecessor in the queue, other people might jump the queue before them such that they would need to wait longer (and thus spend more time on the task). Note that, in contrast to Study 1, the negative consequence of not engaging in the adaptive behavior was thus not solely contingent on the participant’s choice to engage in distancing behavior but also on other people’s behavior (to jump the queue). In this experimental context, however, keeping more distance was directly related to other people jumping the queue.

Participants completed 2 practice trials in which they first saw the shopping item they needed to find (e.g., potatoes). They then saw a picture with shelves in a store and in front of the shelves would be a poster reminding people that during the COVID-19 crisis they should keep 2 m away from others. Different items would then appear and disappear on the shelves and participants needed to click on the item they needed to find. If they were too slow to do so (> 1 s), participants saw a prompt that they were too slow and that someone else took the item before them. When participants clicked on the item in time, they were informed that they could now pay for the item to complete shopping for this item. Participants were then shown a queue for the check out and they were asked where they wanted to stand. There were 5 options with the first option being about 0.5 m away from the final shopper in the queue and each next option 0.5 m further away. After clicking a spot, participants would receive feedback about the distance they had kept from others (place 1: 0.5 m, 2: 1 m, 3:1.5 m, 5:2 m, 5:2.5 m) and how long they needed to wait. If participants chose for the options with more distance (places 2–5), they would see a prompt that other people cut the queue in front of them and how long they therefore needed to wait (closest place: zero seconds, second or third closest place: 10 s, fourth or fifth closest place: 20 s). Information about the exact delay for the different options was not provided beforehand. After this feedback, the next trial would start. After the practice trials, participants completed 6 test trials.

Next, participants completed the same shopping task a second time. Trials were identical with two exceptions. First, participants in the goal inference nudging condition saw a poster near the shelves on which an old couple was presented with the following text: “One person not keeping their distance can lead to the death of hundreds of grandparents, like your own. Make your own choice. What do you choose to do, keep your distance or not?” Second, for participants in the action inference nudging condition, in accordance with often used nudging interventions^56^, there were circles and crosses on the floor at checkout that specified where participants should stand in the queue to keep sufficient distance from others. There was a cross below the first 3 places and a circle indicating 2 m under the fourth spot.

When participants came back for the final part of the study, they first completed demographic questions and two questions about social distancing when shopping in real life that they had also completed during pre-screening. One question asked on how many separate occasions they had thought about keeping their distance and decided to do so while a second question asked how often they might not have kept sufficient distance from people other than the members of their immediate household. Participants then completed the shopping task a third time (identical to Time 1, without the nudges), were probed for demand compliance and reactance and were debriefed and thanked for their participation.

The main analyses constituted a 3 (Intervention Condition) × 3 (Time of Task Performance) mixed analysis of variance (ANOVA) on the mean distance value at checkout in the virtual shopping task and on responses to the real-life social distancing questions and planned t-tests comparing differences between conditions (Table 4).

**Table 4:** Means, SDs, and regression-estimated effects of condition for increases in social distancing behavior and distance violations compared to baseline in Study 2. The table reports the average and standard deviations (in parentheses) of increases in social distancing behavior compared to baseline in Study 2 and the p-value of the effect of condition in an ANOVA for each variable.

	Goal inference nudging	Action inference nudging	Control	p-value
Social distancing shopping task 2 (in meters)	0.2 (0.4)	0.3 (0.4)	0.0 (0.4)	< .001
Social distancing shopping task 3 (in meters)	0.3 (0.5)	0.1 (0.4)	0.1 (0.4)	.032
Social distancing thoughts real-life shopping	8.5%	9.6%	1.3%	.043
Social distancing real-life shopping	1.1 (3.8)	1.0 (2.9)	−0.2 (2.9)	.027
Distance violations real-life shopping	−2.3 (2.7)	1.5 (2.8)	1.2 (2.8)	.058

### Study 3—Participants

We observed hand sanitizing behavior of all customers to a local store on three weekdays (Monday, Tuesday and Thursday) in February 2021. Observation occurred in three two-hour timeslots (9 am–11 am; 12 am–2 pm; 3 pm–5 pm) with each condition assigned to each timeslot once on a randomly determined weekday. There were 2198 customers in total. Target sample size was a minimum of 1200 participants which we estimated to be at least the number of participants during the planned 3 × 2 h observation slots (to allow > 0.95 power to observe a small difference in a proportion test comparing two conditions at alpha = 0.05).

### Study 3—Design

One observer registered whether each participant entered the shop with or without disinfecting their hands. A second observer observed hand sanitization inside the shop at the entrance of the fresh foods area where a second dispenser was placed. At the latter place, the amount of used disinfecting alcohol was also weighed by a third observer. None of the observers were informed about the study hypotheses or conditions. On the final day of the study, a total of 5% of the customers coming out of the shop were probed in a funnel debriefing procedure to examine whether they had noticed the intervention or were aware that they had been observed or had taken part in a study. None of the customers indicated any awareness of the observation study. A total of 60% of the customers in the goal inference nudging condition indicated awareness of the posters or the message.

In the control condition, the alcohol dispenser at the entrance was placed at its original location in the corner of the entrance hall. In the action inference nudging condition*,* the dispenser was placed closer to the entrance door and there was a red sign next to the dispenser indicating: ‘please disinfect hands’, according with often used nudging interventions^57^. The goal inference nudging condition was identical to the action inference nudging condition except that the information on the sign was replaced with the information: ‘Disinfecting hands saves life’s. Will you disinfect your hands?’. Two posters were placed next to the sign which repeated this message in reference to elderly and vulnerable people and showed images of these two groups. There was no nudging at the entrance of the fresh foods area (no difference between the three conditions).

The analysis constituted a mixed effects logistic (and linear) regression with condition as predictor, time of day as control variable, and the proportion of people disinfecting their hands (and the amount of disinfection alcohol used) as dependent variable, and planned proportion (t-) tests comparing differences between the three conditions (Table 5).

**Table 5:** Proportions and means for dependent variables of Study 3. The table reports the proportions, means, and standard deviations (in parentheses) of all dependent variables in Study 3, the p-value of the effect of condition in an ANOVA for each variable, and the number of participants in each condition.

	Goal inference nudging	Action inference nudging	Control	p-value
Proportion disinfectors at entrance	68.1%	66.1%	44.0%	< .001
Proportion disinfectors at generalization site	40.1%	33.7%	32.4%	.012
Amount of disinfectant used	0.48 (0.06)	0.34 (0.04)	0.30 (0.03)	< .001
Number of participants	715	687	796	

## Supplementary Information

Supplementary Information.

## Data Availability

Further information on research design and results is available in the Supplementary Online Material linked to this paper. The authors declare no competing interests. The studies were pre-registered at the Open Science Framework (Study 1: https://osf.io/rb7j8/registrations; Study 2: https://osf.io/8r7hs/registrations; Study 3: https://osf.io/bj2ys/registrations). All raw and processed data are available at the Open Science Framework (Study 1: https://osf.io/rb7j8/; Study 2: https://osf.io/8r7hs/; Study 3: https://osf.io/5t6hy/). The code to replicate the analyses and figures in the manuscript and Supplementary Information is available at the Open Science Framework (Study 1: https://osf.io/rb7j8/; Study 2: https://osf.io/8r7hs/; Study 3: https://osf.io/5t6hy/).
