# Supplementary material for: Nudging societally relevant behavior by promoting cognitive inferences
Source: Sci Rep. 2022 Jun 2;12:9201. doi: 10.1038/s41598-022-12964-1 (PMC9161190; doi:10.1038/s41598-022-12964-1)
Supplement: Supplementary file 1 — Supplementary Information. [file 41598_2022_12964_MOESM1_ESM.docx]

**Supplementary Information for**

Nudging societally relevant behavior by promoting cognitive inferences

Pieter Van Dessel, Yannick Boddez, Sean Hughes

Correspondence to: Pieter Van Dessel

Email: Pieter.VanDessel@UGent.be

**This PDF file includes:**

Extended Materials and Methods

Extended Results

Extended figures

Deviations from pre-registration

**Extended Materials and Methods**

### Study 1 - Participants

We recruited 350 participants via via the Prolific Academic Research website (<https://prolific.ac>). Only UK participants whose first language was English were allowed to participate. The sample size of 350 completed contributions was determined based on an a priori power analysis such that we would have sufficient power (i.e., power > 0.90) to detect a between-subjects effect of *d* = 0.40 with an alpha criterion p < .05 in a one-tailed between-subjects *t-*test comparing scores between two conditions taking into account that some participants would not come back for the Phase 2 part of the experiment. The effect size of *d* = 0.40 was chosen based on previous studies with inference training which have typically shown effects bigger than *d* = 0.40 compared to control conditions.

Prior to data-collection, target sample size was pre-registered together with the study design and data-analytic plans. The pre-registered plans, raw data, experimental and analytic scripts are available at <https://osf.io/8dfe3/>. We also pre-registered the following hypotheses: For the slider value score, we predicted (1) the T1 score to be higher than the T2 score for the action and goal inference nudging conditions (H1, H2), (2) the T1 score to be higher than the T3 score for the goal inference nudging condition (H3), (3) difference scores from T1 to T2 to be bigger for both the action and the goal inference nudging conditions compared to the control condition (H4, H5), and (4) difference scores from T1 to T3 to be bigger for the goal inference nudging compared to the control condition and compared to the action inference nudging condition (H6, H7). For the self-reported environment-friendly behavior scores, we predicted (1) the T1 scores to be lower than the T3 scores for the goal inference nudging condition (H8a, H8b), and (2) difference scores from T1 to T3 to be lower for the goal inference nudging compared to the control condition and compared to the action inference nudging condition (H9a, H9b; H10a, H10b).

Following our pre-registered data analysis plan, we excluded the data from participants who (a) did not fully complete all questions and tasks at Phase 1 (12 participants; i.e., 3.4%), (b) did not complete Phase 2 (36 participants; i.e., 10.3%), or (c) indicated issues performing the experiment (1 participant; i.e., 0.3%). Analyses were performed on the data of 301 participants (217 women, mean age = 36, *SD* = 12).

### Study 1 - Design

The study constituted a mixed design with one between-subjects factor with 3 levels: Intervention Condition (goal inference nudging condition, action inference nudging condition, control condition). Participants were randomly assigned to the different interventions. There was also one within-subjects factor with 3 levels: Time of Task performance (before the writing task: Time 1; after the writing task: Time 2; 24 hours after Phase 1: Time 3).

In line with standard recommendations to prevent selective attrition (Zhou & Fishbach, 2016), participants were first (1) informed about the study duration and the requirement to complete different phases and (2) asked to do their best to complete all tasks in a thoughtful manner without taking a break to help facilitate scientific progress. Participants were also asked not to complete the study if they were color blind. Participants then provided informed consent and indicated their age, gender, and identification number for payment purposes. Next, participants were asked whether, in the previous 24 hours, they ever considered that they should try to limit their energy consumption such as by conserving electricity (response options: yes/no) and on how many separate occasions they acted in accordance with this thought (e.g., thinking that closing the fridge or turning of the lights saves energy and actually doing so). They also indicated to what extent they found it personally important to (1) act environmental friendly and (2) make as much money as possible in the studies they perform on Prolific Academic (see Table S1 for descriptives and randomization checks).

Participants then received instructions about the figure search task. Participants were informed that they would need to find specific colored figure as quickly as possible and that they would receive more money than the default study payment if they would be faster than other participants in this task (for verbatim instructions see Supplementary Material available at OSF Project link). Instructions then specified that the researchers are aware that presenting bright colors and large figures consumes a lot of electricity (a link was provided to a website supporting this claim) and that participants would therefore be allowed to decide for themselves how brightly they wanted to present the figures. To this end, they could move a slider to the left (and save more energy) or to the right (and save less energy). We explained that participants thus could serve two goals by use of the slider: to save more electricity but also make the task more difficult or to save less electricity but also make the task easier. After completing an instruction check, participants completed 8 practice trials in which they saw a display with 140 figures (circles and squares) in 4 different colors (blue, green, pink, brown) and they needed to click on one specific figure (e.g., a blue square) as quickly as possible (Extended Figure 1). A slider was presented that participants could move left to display the figures less brightly and save more energy or to the right to display the figures more brightly (slider values 0 – 100; slider start value = 50). After clicking on the figure, participants were informed about their response time. After the practice phase, participants received the generic feedback that they were a little slower than the average participant who did this task before them. They then completed 40 test trials.

After the first figure search task, participants in the goal inference nudging condition were informed that there are many harmful effects on the environment that result from wasting electricity. They were asked to list negative (positive) thoughts and consequences that would come to mind if they or others would choose energy wasteful (energy saving) behavior. Finally, participants selected what type of behavior they thought they would be more likely to choose to emit in the future given the consequences of energy saving and energy wasteful behavior they just stipulated themselves. Participants in the control and action inference nudging condition performed the same task about an unrelated topic (drug use).

Participants then completed the same 40 test trials for the second figure search task with one exception. For participants in the action inference nudging condition, there were three changes. In accordance with other nudging interventions (Sussman & Gifford, 2012), (1) a cue was presented: a large green arrow with the text: “Move slider to the left to be more environmentally friendly!”, (2) the slider value was presented in green/orange/red depending on the value, and (3) the slider value was set lower (at 75 rather than 50) at the start of the task. Participants could then note any (visibility or personal) problems they had with the study and were reminded to come back for the second part of the study the following day.

The next day, participants first completed the same demographic questions and questions about real-life energy saving behavior as before and then completed the third figure search task (identical to the first figure search task). Next, participants were probed for demand compliance and reactance and were debriefed and thanked for their participation.

The main analyses constituted a 3 (Intervention Condition) x 3 (Time of Task Performance) mixed analysis of variance (ANOVA) on the mean figure search task slider values and real-life energy saving behavior and planned t-tests comparing differences in slider values and real-life energy saving behavior between conditions (Data Table S2).

### Study 2 - Participants

In May 2020, during the first COVID-19 lockdown in the UK, we recruited 250 UK volunteers via Prolific Academic to allow sufficient power to find a between-subjects effect of *d* = 0.40. Invitation to the study was based on a pre-screening study in which the targeted participants had indicated that (1) they would go shopping later that day, (2) they would be available to complete the first study part before going shopping and the second part after going shopping, (3) the last time they went shopping was less than a week ago, (4) they had not kept their distance at least twice during the last time they went shopping, (5) they found it important that their grandparents would stay healthy, (6) they did not find it easy to adhere to COVID-19 guidelines, (7) they did not find it very important to follow COVID-19 guidelines, and (8) they found it important to make money on Prolific.

We pre-registered the following hypotheses: For the shopping task distance keeping score (higher = more distance), we expected that (1) the score on T1 would be lower than the score on T2 for both the behavior and goal inference nudging conditions (H1, H2), (2) the score on T1 would be lower than the score on T3 for the goal inference nudging condition (H3), (3), difference scores from T1 to T2 (T2 score – T1 score) would be bigger for the action inference nudging compared to the control condition and for the goal inference nudging compared to the control condition (H4, H5), and (4) difference scores from T1 to T3 (T3 score – T1 score) would be bigger for the goal inference nudging compared to the control condition and compared to the action inference nudging condition (H6, H7). For the self-reported real-life distance keeping scores, we expected that (1) the thought score on T1 would be lower and the distance keeping violation score on T1 would be higher than the score on T3 for the goal inference nudging condition (H8, H9), (2) difference thought scores from T1 to T3 (T3 score – T1 score) would be higher and difference distance keeping violation scores from T1 to T3 would be lower for the goal inference nudging compared to the control condition and compared to the action inference nudging condition (H10a,b, H11 a,b).

Following our pre-registered data analysis plan, we excluded the data from participants who (a) did not pass the attention check during Phase 1 (28 participants; i.e., 10.3%), or (b) indicated issues performing the experiment (0 participants). Analyses were performed on the data of 222 participants (125 women, mean age = 25, *SD* = 4). Table S3 provides descriptives and randomization checks.

### Study 2 – Design

**Pre-screening study.** A total of 1872 volunteers were recruited from Prolific Academic to participate in a research study that took place at the end of May 2020, when the first corona virus lockdown took place in the UK with government recommendations to stay at home as much as possible, limit contact with other people and keep distance 2 meters apart from other people when going out. Participants could only perform this experiment if they indicated in their pre-screening questionnaire on Prolific that (1) their first language is English, (2) they live in the UK and (3) between 20 and 30 years (young people show more trouble in adhering to the corona virus regulations).

The study was said to be specifically for people who found it difficult to follow the UK corona virus guidelines for social distancing, who would do shopping later that day, and who would be OK with answering questions in relation the Covid-19 epidemic. Participants were asked not to complete the study if this was not the case for them. Participants were asked to report age and gender and were then asked whether (1) they would go out of their house that day to do shopping in a place where there would be people other than the members of their immediate household, (2) how many days it had been since they had last went out to do shopping, and (3) whether they would be available later that day to perform a study on Prolific before and after they did their shopping (response options: ‘yes’, ‘no’, ‘unsure’). Participants were also asked whether they found it difficult to adhere to UK government recommendations for social distancing due to the corona crisis. Responses to this question were provided on a 7-point Likert scale ranging from 0 (not at all) to 7 (very much) with 4 (moderately) as a midpoint.

For use in the main study, we probed real-life social distancing behavior by asking participants to think back to when they last did shopping and report whether, at that time, (1) they ever considered that they should keep their distance from other people to at least 2 meters (yes/no) (real-life distance keeping thoughts), and (2) if so, on how many separate occasions they not only had this thought but also acted in accordance with it (e.g., thinking that they should move further away and actually doing so) (real-life distance keeping score). Next, participants were asked to give a rough estimate of the number of separate occasions during their last time shopping in which they (intentionally or unintentionally) might NOT have kept their distance from others to at least 2 meters (real-life distance keeping violation score).

Participants also indicated to what extent they found it personally important to (1) follow recommendations for social distancing, (2) make as much money as possible in the experiments they perform on Prolific Academic, and (3) to have their grandparents stay heathy (7-point Likert scale).

**Main study.**

The study constituted a mixed design with one between-subjects factor with 3 levels: Intervention Condition (goal inference nudging condition, action inference nudging condition, control condition). Participants were randomly assigned to the different interventions. There was also one within-subjects factor with 3 levels: Time of Task performance (before the intervention: Time 1; during the intervention: Time 2; after shopping in real-life: Time 3).

After informing participants about the duration of the study, they received instructions about the virtual shopping task. Participants were also asked to imagine that they themselves were shopping during the COVID-19 crisis and that they wanted to complete their shopping quickly. They were informed that performing this task faster than other participants would lead to a bigger monetary reward and that, each trial, they would first need to acquire a specific item in a virtual store by clicking on it and then queue at checkout to pay for the item. At checkout they would need to select where to stand in the queue for checkout by clicking on an open place but if they would choose to stand farther away from their predecessor in the queue, other people might jump the queue before them such that they would need to wait longer (and thus spend more time on the task).

Participants completed 2 practice trials in which they first saw the shopping item they needed to find (e.g., potatoes). They then saw a picture with shelves in a store and in front of the shelves would be a poster reminding people that during the COVID-19 crisis they should keep 2 meters away from others (Extended Figure 2). Different items would then appear and disappear on the shelves and participants needed to click on the item they needed to find. If they were too slow to do so (>1 second), participants saw a prompt that they were too slow and that someone else took the item before them. When participants clicked on the item in time, they were informed that they could now pay for the item to complete shopping for this item. Participants were then shown a queue for the check out and they were asked where they wanted to stand. There were 5 options with the first option being about 0.5 meters away from the final shopper in the queue and each next option 0.5 meters further away. After clicking a spot, participants would receive feedback about the distance they had kept from others (place 1: 0.5 meters, 2: 1 meter, 3:1.5 meters, 5:2 meters, 5:2.5 meters) and how long they needed to wait. If participants chose for the options with more distance (places 2-5), they would see a prompt that other people cut the queue in front of them and they therefore needed to wait longer (closest place: zero seconds, second or third closest place 10 seconds, fourth or fifth closest place: 20 seconds). Subsequently, the next trial would start. After the practice trials, participants completed 6 test trials.

Next, participants completed the same shopping task a second time. Trials were identical with two exceptions. First, participants in the goal inference nudging condition saw a poster near the shelves on which an old couple was presented with the following text: “One person not keeping their distance can lead to the death of hundreds of grandparents, like your own. Make your own choice. What do you choose to do, keep your distance or not?” Second, for participants in the action inference nudging condition, in accordance with often used nudging interventions (Crymble, 2020), there were circles and crosses on the floor at checkout that specified where participants should stand in the queue to keep sufficient distance from others. There was a cross below the first 3 places and a circle indicating 2 meters under the fourth spot.

When participants came back for the final part of the study, they first completed demographic questions and two questions about social distancing when shopping in real life that they had also completed during pre-screening. One question asked on how many separate occasions they had thought about keeping their distance and decided to do so while a second question asked how often they might not have kept sufficient distance from people other than the members of their immediate household. Participants then completed the shopping task a third time (identical to Time 1, without the nudges), were probed for demand compliance and reactance and were debriefed and thanked for their participation.

The main analyses constituted a 3 (Intervention Condition) x 3 (Time of Task Performance) mixed analysis of variance (ANOVA) on the mean distance value at checkout in the virtual shopping task and on responses to the real-life social distancing questions and planned t-tests comparing differences between conditions (Table S4).

### Study 3 - Participants

We observed hand sanitizing behavior of all customers to a local store on three weekdays (Monday, Tuesday and Thursday) in February 2021. Observation occurred in three two-hour timeslots (9am-11 am; 12am-2pm; 3pm-5pm) with each condition assigned to each timeslot once. There were 2198 customers in total. Target sample size was a minimum of 1200 participants which we estimated to be at least the number of participants during the planned 3 x 2 hour observation slots (to allow sufficient power (>0.95) to observe a small difference in a proportion test comparing two conditions at alpha = .05.

### Study 3 - Design

One observer registered whether each participant entered the shop with or without disinfecting their hands. A second observer observed hand sanitization inside the shop at the entrance of the fresh foods area where a second dispenser was placed. At the latter place, the amount of used disinfecting alcohol was also weighed by a third observer. All observers switched positions between the different timeslots. Because the dispenser always dispenses a specified amount of disinfecting alcohol, this allowed us to calculate a (conservative) index of interrater reliability for all raters. The interrater reliability was good, *r*(7) = 0.77. None of the observers were informed about the study hypotheses or conditions. On the final day of the study, a total of 5% of the customers coming out of the shop were probed in a funnel debriefing procedure to examine whether they had noticed the intervention or were aware that they had been observed or had taken part in a study. None of the customers indicated any awareness of the observation study. A total of 60% of the customers in the goal inference nudging condition indicated awareness of the posters or the message.

In the control condition, the alcohol dispenser at the entrance was placed at its original location in the corner of the entrance hall. In the action inference nudging condition*,* the dispenser was placed closer to the entrance door and there was a red sign next to the dispenser indicating: ‘please disinfect hands’, according with often used nudging interventions (Aarestrup & Moesgaard, 2016). The goal inference nudging condition was identical to the action inference nudging condition except that the information on the sign was replaced with the information: ‘Disinfecting hands saves life’s. Will you disinfect your hands?’. Two posters were placed next to the sign which repeated this message in reference to elderly and vulnerable people and showed images of these two groups (Extended Figure 3). There was no nudging at the entrance of the fresh foods area (no difference between the three conditions).

The analysis constituted a mixed effects logistic (and linear) regression with condition as predictor, time of day as control variable, and the proportion of people disinfecting their hands (and the amount of disinfection alcohol used) as dependent variable, and planned proportion (t-) tests comparing differences between the three conditions (Table S5).

**Extended Results**

### Study 1 - Results

**Participant characteristics.** T-tests and proportion tests comparing age, gender, Time 1 energy saving behavior and Time 1 goals for participants in the three conditions, did not reveal any significant differences, *t*s < 1.87, *p*s > .063. Notably, a Wald-test revealed a significant difference in the proportion of participants reporting energy saving behavior at Time 1, *χ ^2^*(2) = 6.30, *p* = .043. Specifically, the proportion of participants reporting energy saving behavior at Time 1 was higher for the goal inference nudging condition than for the control condition, *χ ^2^*(1) = 4.68, *p* = .030.

**Search Task.** Slider values were reversed such that higher scores indicate more energy saving behavior. We performed a 3 (Intervention Condition) x 3 (Time of Task Performance) mixed analysis of variance (ANOVA) on the mean slider values. The ANOVA revealed a main effect of Intervention Condition, *F*(2,298) = 5.29, *p* = .006, a main effect of Time of Task Performance, *F*(1.74, 519.30) = 12.81, *p* < .001, and a significant interaction, *F*(3.49,519.30) = 5.13, *p* < .001.

In-line with hypotheses H1 and H2, the mean T2 slider value was higher than the T1 value for both the action inference (T1: *M* = 33.28, *SD* = 19.32; T2: *M* = 37.22, *SD* = 23.69), *t*(95) = 1.81, *p* = .037, *d* = 0.18, and goal inference nudging conditions (T1: *M* = 37.54, *SD* = 19.70; T2: 47.71, *SD* = 22.47), *t*(93) = 5.33, *p* < .001, *d* = 0.55. For the control condition, the mean T2 slider value did not significantly differ from the T1 value (T1: *M* = 36.21, *SD* = 19.64; T2: *M* = 36.26, *SD* = 20.29), *t*(110) = -0.03, *p* = .97, *d* = 0.00. In line with hypothesis H5, the increase in mean slider value from T1 to T2 was bigger for the goal inference nudging compared to the control condition, *t*(203) = 4.31, *p* < .001, *d* = 0.60. In contrast, hypothesis H4, indicating that he increase in mean slider value from T1 to T2 was bigger for the action inference nudging compared to the control condition, was not supported by the data, *t*(205) = 1.53, *p* = .064, *d* = 0.21.

In-line with hypothesis H3, the mean T3 slider value was higher than the T1 value for the goal inference nudging condition (T1: *M* = 37.54, *SD* = 19.70; T3: 44.22, *SD* = 20.03), *t*(93) = -3.68, *p* < .001, *d* = 0.38. For the control and action inference nudging conditions, the mean T3 slider value did not significantly differ from the T1 value, *t*s < 0.90*, ps* > .37 *, ds* < 0.10. In line with hypothesis H6 and H7, the increase in mean slider value from T1 to T3 was bigger for the goal inference nudging compared to the control condition and compared to the action inference nudging condition, *t*s > 2.03*, ps* < .022 *, ds* > 0.29.

**Environment-friendly scores.** In contrast with hypothesis H8a, a test comparing the proportion of participants in the goal inference nudging condition who indicated engaging in environment-friendly behaviour did not reveal a lower proportion at T1 compared to T3 (T1: 60.63%; T3: 71.28%), γ^2^(1) = 1.92, *p* = .083, cohen’s *h* = 0.23. We also did not observe significant differences in proportions for the action inference nudging condition, (T1: 75.68%; T3: 66.67%), γ^2^(1) = 1.78, *p* = .18, cohen’s *h* = -0.20, or the control condition, (T1: 73.96%; T3: 63.54%), γ^2^(1) = 1.96, *p* = .16, cohen’s *h* = -0.23. In line with hypothesis H9a and H10a, the increase in proportions from T1 to T3 was bigger for the goal inference nudging compared to the action inference nudging and compared to the control condition, *t*s > 2.82*, ps* < .003 *, ds* > 0.39.

In contrast with hypotheses H8b, the mean number of environment-friendly behaviors was not significantly lower at T1 than at T3 for the goal inference nudging condition participants (T1: *M* = 2.71, *SD* = 4.83; T2: M = 2.62, *SD* = 3.24), *t*(93) = 0.10, *p* = .57, *d* = 0.02. Unexpectedly, the mean number of environment-friendly behaviors was significantly *higher* at T1 than at T3 for both the action inference nudging (T1: *M* = 2.36, *SD* = 3.08; T2: 1.72, *SD* = 2.27), *t*(95) = 2.39, *p* = .019, *d* = 0.24, and the control condition participants (T1: *M* = 2.82, *SD* = 2.84; T2: 2.00, *SD* = 2.43), *t*(110) = 3.16, *p* = .002, *d* = 0.30. In contrast with hypotheses H9b and H10b, goal inference nudging participants did not exhibit a significantly stronger increase in environment-friendly behavior compared to the action inference nudging, *t*(188) = 0.93, *p* = .18, *d* = 0.13, or the control condition participants, *t*(205) = 1.28, *p* = .10, *d* = 0.18.

**Exploratory questions.** Reactance scores (*M* = 1.36, *SD* = 0.89) and demand compliance scores (*M* = 1.58, *SD* = 1.07) were low (i.e., significantly lower than the mid-point of the scale), *p*s < .001, and t-tests did not reveal differences between conditions, *ts <* 0.53, *p*s > 59. Believability of the performance-based money (*M* = 4.33, *SD* = 1.96) and of the environment-friendliness of using the slider (*M* = 3.22, *SD* = 1.83) was moderate (i.e., slightly higher than the mid-point of the scale for the performance-based money and slightly lower than the mid-point of the scale for the environment-friendliness). T-tests revealed higher believability for the environment-friendliness in the inference compared to the action inference nudging condition, *t*(188) = 2.48, *p* = .014, but no other significant differences, *t*s < 1.60*, ps* > .11.

### Study 2 - Results

**Participant characteristics.** T-tests comparing age, gender, and Time 1 goals for participants in the three conditions, did not reveal any significant differences, *t*s < 1.78, *p*s > .078.

**Shopping Task.** We performed a 3 (Intervention Condition) x 3 (Time: Task Performance at T1, T2, T3) mixed analysis of variance (ANOVA) on the mean distance scores. The ANOVA revealed a main effect of Time, *F*(1.95,427.41) = 28.76, *p* < .001, and a significant interaction of Intervention Condition x Time, *F*(3.49,519.30) = 6.76, *p* < .001.

In-line with hypotheses H1 and H2, the mean T1 distance score was lower than the T2 score for both the action inference (T1: *M* = 0.92, *SD* = 0.47; T2: *M* = 1.19, *SD* = 0.63), *t*(77) = -6.37, *p* < .001, *d* = 0.72, and goal inference nudging conditions (T1: *M* = 0.93, *SD* = 0.46; T2: 1.10, *SD* = 0.60), *t*(70) = -3.34, *p* < .001, *d* = 0.40. For the control condition, the mean T2 distance score did not significantly differ from the T1 score (T1: *M* = 0.91, *SD* = 0.44; T2: *M* = 0.94, *SD* = 0.56), *t*(72) = -0.87, *p* = .39, *d* = 0.10. In line with hypothesis H4 and H5, the increase in distance score from T1 to T2 was bigger for the action inference nudging compared to the control condition, *t*(149) = 4.18, *p* < .001, *d* = 0.68, and for the goal inference nudging compared to the control condition, *t*(142) = 2.16, *p* = .016, *d* = 0.36. A two-tailed *t*-test revealed no significant difference between action inference nudging and goal inference nudging difference scores, *t*(147) = 1.59, *p* = .11, *d* = 0.26.

In-line with hypothesis H3, the mean T1 distance score was lower than the T3 score for the goal inference nudging condition (T3: M = 1.22, *SD* = 0.61), *t*(70) = -5.00, *p* < .001, *d* = 0.59. For the control and action inference nudging conditions, the mean T1 distance score was also lower than the T3 score (control condition: *t*(93) = -2.80, *p* = .003, *d* = 0.33; action inference nudging condition: *t*(93) = -3.40, *p* = .003, *d* = 0.36). In line with hypothesis H6 and H7, the increase in distance keeping score from T1 to T3 was bigger for the goal inference nudging compared to the control condition and compared to the action inference nudging condition, *t*s > 1.95*, ps* < .027 *, ds* > 0.31. A two-tailed *t*-test revealed no significant difference between action inference nudging and control difference scores, *t*(149) = 0.47, *p* = .32, *d* = 0.08.

**Real-life behavior scores.** The ANOVA on real-life distance keeping scores revealed a main effect of Time, *F*(1,219) = 8.79, *p* < .001, and a significant interaction of Intervention Condition x Time, *F*(2,219) = 3.69, *p* =.027. In accordance with hypotheses H8, the mean distance keeping score was lower at T1 than at T3 for the goal inference nudging condition (T1: *M* = 4.15, *SD* = 2.90; T2: 5.28, *SD* = 4.48), *t(*70) = 2.47, *p* = .008, *d* = 0.29. The mean score was not significantly lower at T1 than at T3 for the control condition, *t*(72) = -0.57, *p* = .71, *d* = 0.07, but it was for the action inference nudging condition, *t(*77) = 2.99, *p* = .002, *d* = 0.34. In accordance with hypotheses H10a but in contrast with hypothesis H10b, the increase in scores was higher for the goal inference nudging compared to the control condition, *t*(142) = 2.34, *p* = .010, *d* = 0.39, but not compared to the action inference nudging condition, *t*(147) = 0.23, *p* = .41, *d* = 0.04.

The ANOVA on real-life distance keeping violation scores revealed a main effect of Time, *F*(1,219) = 77.02, *p* < .001, but not a significant interaction of Intervention Condition x Time, *F*(2,219) = 2.88, *p* = .058. In accordance with hypotheses H9, the mean distance keeping violation score was higher at T1 than at T3 for the goal inference nudging condition (T1: *M* = 5.24, *SD* = 4.05; T2: *M* = 2.99, *SD* = 3.35), *t(*70) = 6.92, *p* < .001, *d* = 0.82. The mean score was also significantly higher at T1 than at T3 for control condition, *t*(72) = 3.57, *p* < .001, *d* = 0.42, and for the action inference nudging condition, *t(*77) = 4.73, *p* < .001, *d* = 0.54. In accordance with hypotheses H11a and H11b, the decrease in violation scores was higher for the goal inference nudging compared to the control and action inference nudging conditions, *ts >* 1.72, *ps* < .044, *ds* > 0.28.

**Exploratory analyses.** A multinomial ANOVA on differences in proportion of participants who indicated distance keeping thoughts at T1 compared to T3 revealed a main effect of Intervention condition, γ^2^(4) = 9.83, *p* = .043. A proportion test revealed a lower proportion at T1 compared to T3 for the goal inference nudging condition (T1: 90.14%; T3: 98.60%), γ^2^(1) = 2.67, *p* = .034, cohen’s *h* = 0.40, but not for the other two conditions, γ^2^s < 2.68, *p*s > .10. We observed a significant difference in proportion difference scores for the inference compared to the action inference nudging condition, *t*(90.55) = 2.01, *p* = .024, *d* = 0.34, but not for the goal inference nudging compared to the control condition, *t*(138.27) = 0.22, *p* = .59, *d* = 0.04.

Reactance scores (*M* = 1.77, *SD* = 1.34) and demand compliance scores (*M* = 1.68, *SD* = 1.11) were low (i.e., significantly lower than the mid-point of the scale), *p*s < .001. T-tests revealed only the difference that action inference nudging and goal inference nudging conditions produced slightly higher demand compliance scores than control, *p*s <.047. Excluding highly reactant and highly demand compliant participants did not change the pattern of results.

ANOVAs on goal scores revealed effects of Time such that they reported a stronger goal to follow recommendations for social distancing at T3 than at T1 and a weaker goal to make as much money as possible in the experiments they perform on Prolific Academic at T3 than at T1. No effects of Time x Intervention were observed.

### Study 3 - Results

**Hand disinfection at the entrance.** In total, 68% of customers in the goal inference nudging condition disinfected their hands, 66% in the action inference nudging condition, and 44% in the control condition. In accordance with our hypotheses, the differences between the nudging conditions and the control condition were significant, revealing a 50-55% increase in the total number of hand disinfectors, *p*s<.001, ORs>1.50.

**Hand disinfection at the fresh foods area.** In total, 40% of the customers entering the fresh foods area disinfected their hands in the goal inference nudging condition, compared to 32% in de action inference nudging condition and 30% in the control condition. In accordance with our hypotheses, there were more disinfectors in the goal inference nudging than in the action inference nudging and control conditions with a 27-33% increase in the total number of hand disinfectors), *p*s < .013, ORs > 1.20^[[1]](#footnote-1)^. We observed concordant effects for the amount of hand disinfectant used. The amount of alcohol per customer entering the fresh foods area was 0.48g in the goal inference nudging condition compared to 0.30g in the action inference nudging condition and 0.34g in the control condition. This amount was significantly higher for the goal inference nudging condition than for both other conditions, *p*s < .016, *d*s > 2.76.

**Extended Figures**


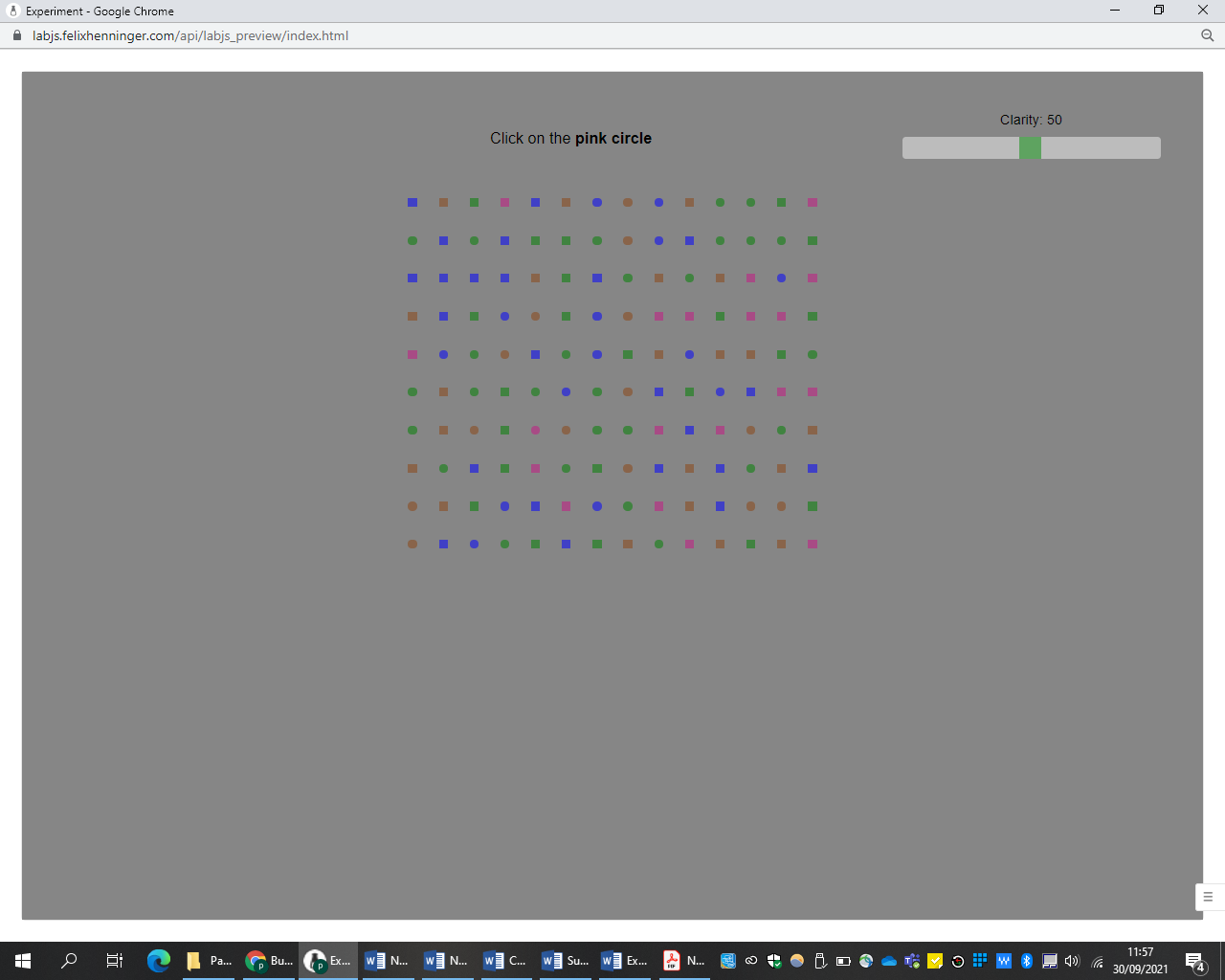

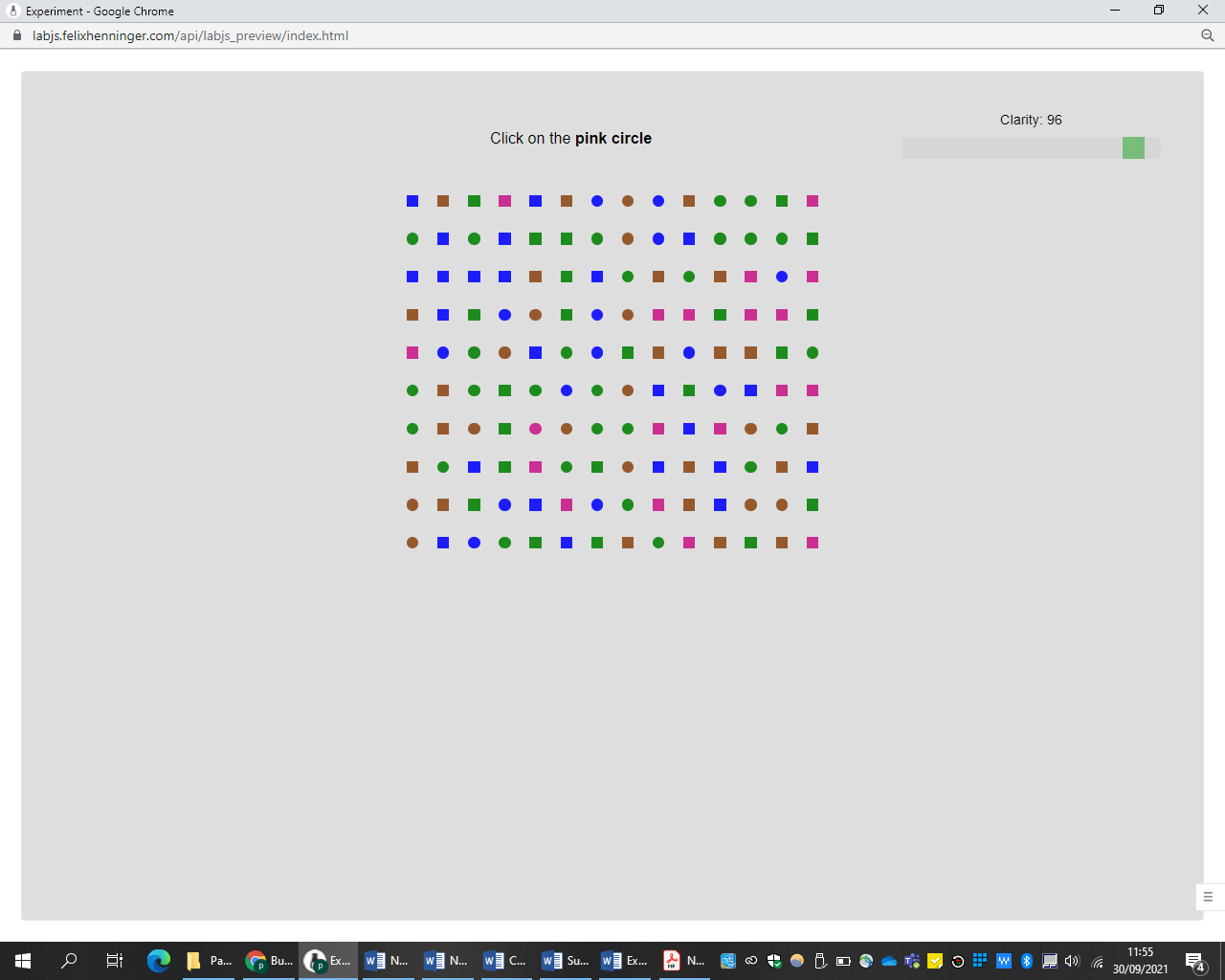


**Extended Figure 1. Illustration of a trial of the figure search task in Study 1 with a lower (left panel) and higher slider value (right panel).** In the figure search task, participants needed to quickly click a specific figure in a display with 140 figures. They could move a slider right (higher slider value) to increase clarity of the figures and display (but waste more electricity) or left to reduce clarity of the figures and display (but save more electricity).


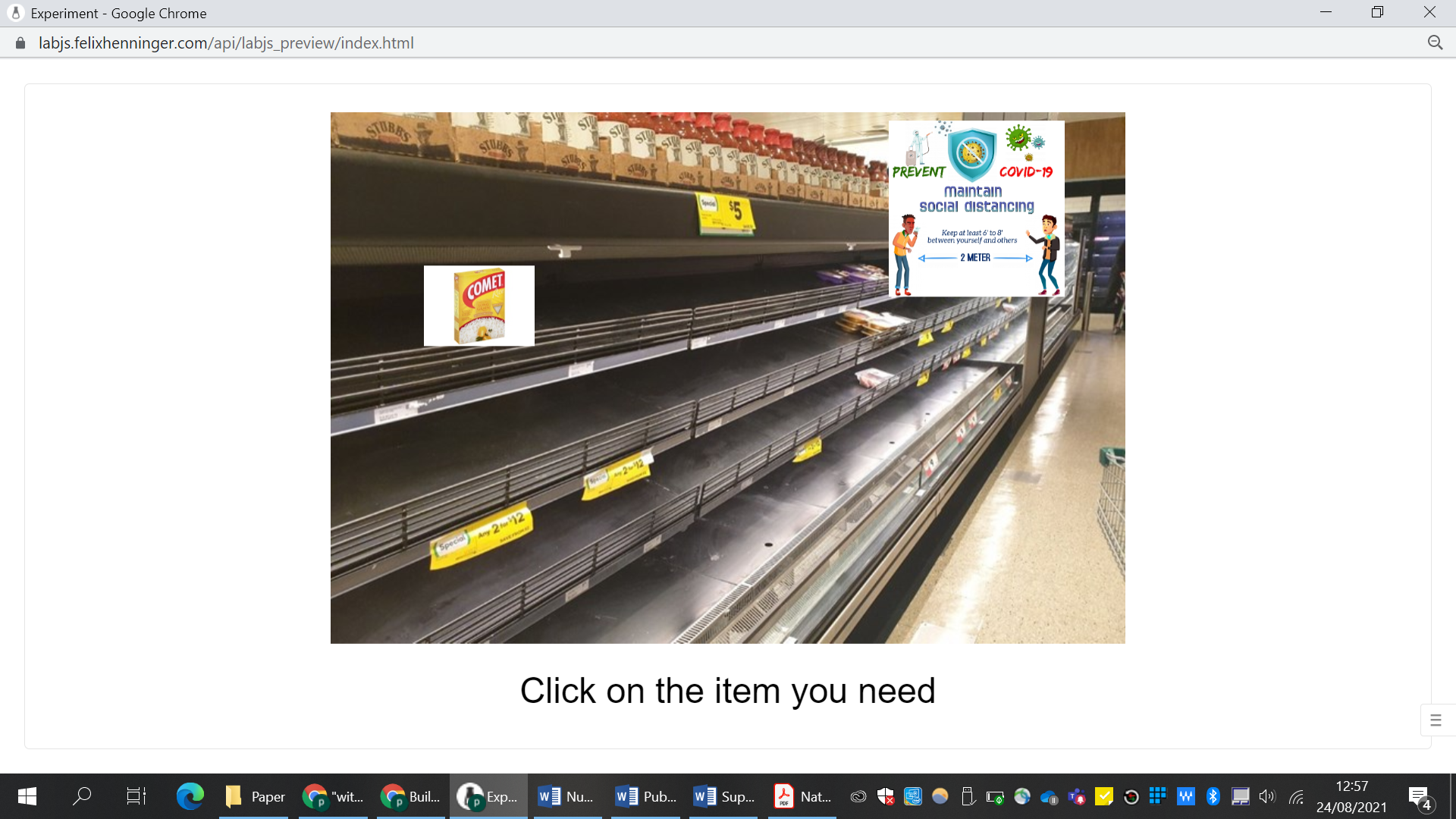

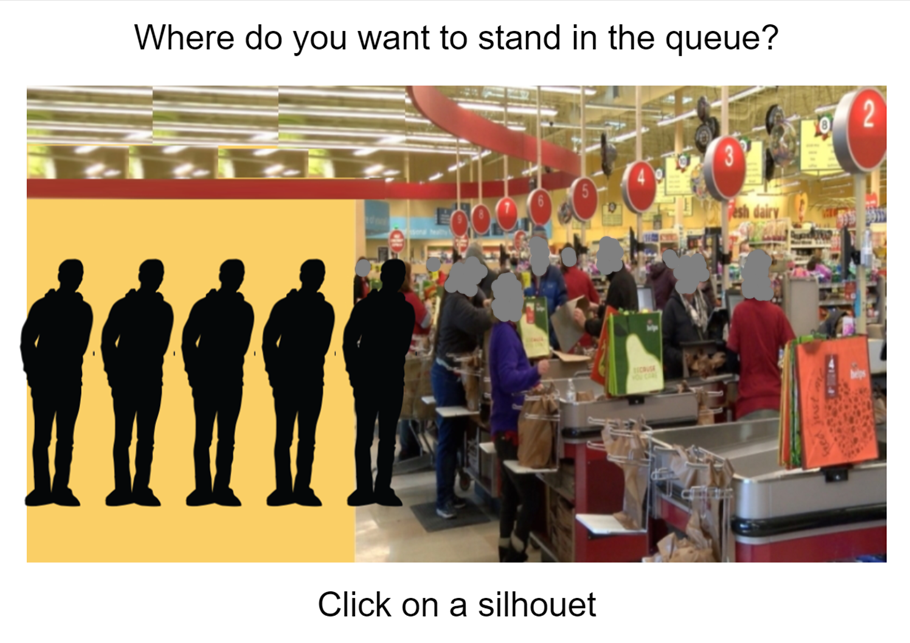


**Extended Figure 2. Illustration of a trial of the virtual shopping task in Study 2.** In the shopping task, participants would see products appear in the shelves (left panel) and needed to quickly click the products. After doing so, they needed to choose where to stand in the queue for check-out (measure of social distancing). Note that, for the sake of anonymity, the image to the right was adapted in the supplementary material so that faces of people in the image were invisible.


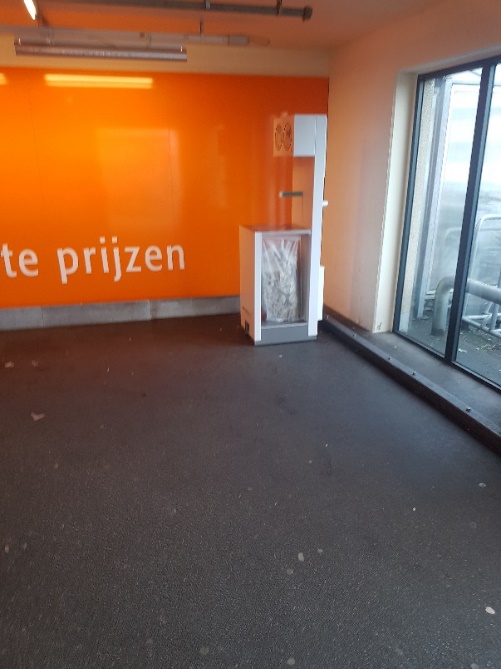

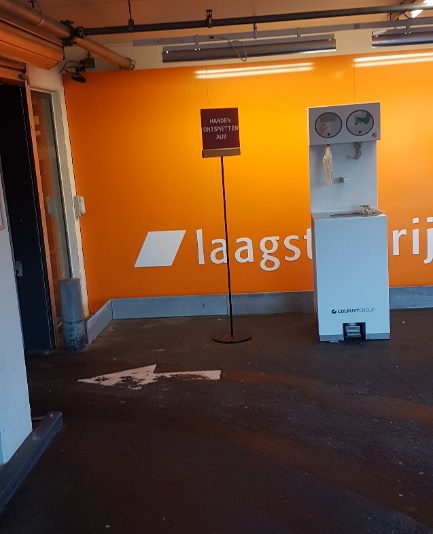

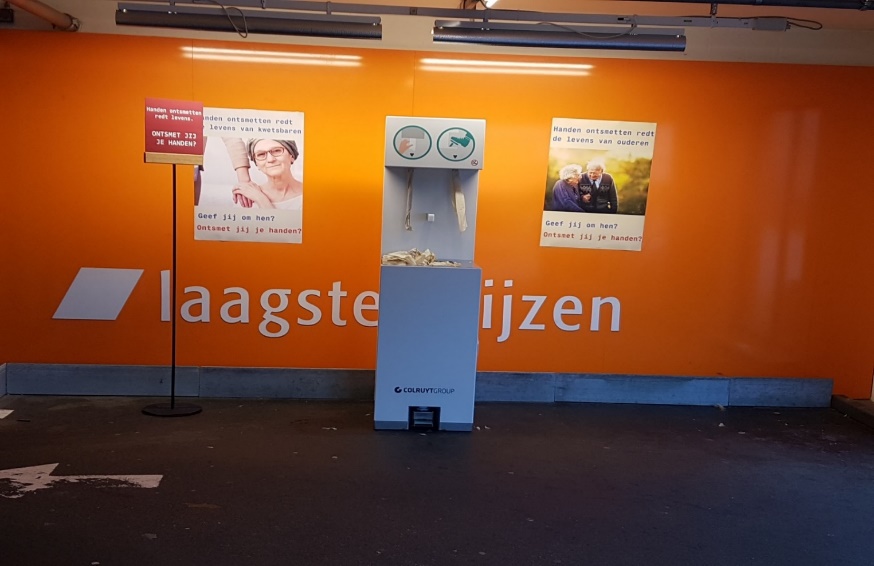


**Extended Figure 3. The environment of the hand disinfection unit at the entrance.** This figure displays the hand disinfection unit at the entrance of the store in the control (left), action inference nudging (middle), and goal inference nudging (right) condition.

**Deviations from preregistration**

The analyses were performed in accordance with the preregistration data analysis plan. However, there are some slight changes from the preregistration for the different studies. First, overall, in the manuscript we talk about action inference nudging (rather than behavior nudging in the preregistration) and goal inference nudging (rather than inference nudging in the preregistration). Second, for Study 1 and 2 we do not report Bayes Factors for the t-tests because we had no informative prior for the analyses and so the Bayes Factors might not be very accurate. Third, we also performed analyses to test differences between participant characteristics (see above: results: participant characteristics). Fourth, we also performed exploratory analyses (see above: results: exploratory analyses).

1. Note that the stronger effect of goal inference nudging compared to action inference nudging is not necessarily due to stronger effects of the goal inference nudge per se (the poster) but could also result from the specific combination of this nudge with the different types of visual and verbal stimuli that were also present in the action inference nudging condition. [↑](#footnote-ref-1)
